# The Presence of Triclosan in Human Hair Samples in Poland—A Pilot Study

**DOI:** 10.3390/ijerph19073796

**Published:** 2022-03-23

**Authors:** Slawomir Gonkowski, Manolis Tzatzarakis, Elena Vakonaki, Krystyna Makowska, Aristidis M. Tsatsakis, Joanna Wojtkiewicz

**Affiliations:** 1 Department of Clinical Physiology, Faculty of Veterinary Medicine, University of Warmia and Mazury in Olsztyn, Oczapowskiego 13, 10-957 Olsztyn, Poland; slawomir.gonkowski@uwm.edu.pl; 2 Laboratory of Toxicology, School of Medicine, University of Crete, 71003 Heraklion, Crete, Greece; tzatzarakis@uoc.gr (M.T.); evakonaki@med.uoc.gr (E.V.); tsatsaka@uoc.gr (A.M.T.); 3 Department of Clinical Diagnostics, Faculty of Veterinary Medicine, University of Warmia and Mazury in Olsztyn, Oczapowskiego 14, 10-957 Olsztyn, Poland; krystyna.makowska@uwm.edu.pl; 4 Department of Physiology and Pathophysiology, School of Medicine, Collegium Medicum, University of Warmia and Mazury, 10-900 Olsztyn, Poland

**Keywords:** biomonitoring, endocrine disruptors, hair, triclosan

## Abstract

Triclosan (TCS) is an organic substance showing antibacterial action, which is commonly used in many branches of industry, including, among others, cosmetics, pharmaceuticals and the food industry. TCS may penetrate into living organisms and negatively affect the health of humans and animals. The majority of previous investigations on TCS biomonitoring in humans have been performed on urine, but currently, studies on hair samples are becoming increasingly important. The aim of this study was to evaluate TCS concentration levels in residents of Olsztyn, a city in northeastern Poland, using a liquid chromatography-mass spectrometry technique. The presence of TCS was observed in 96.7% of samples tested, with concentration levels from 37.9 pg/mg to 3386.5 pg/mg. The mean concentration level of TCS in the present study was 402.6 (±803.6) pg/mg, and the median value was 103.3 pg/mg. Although there were some differences in TCS concentration levels between males and females, humans of various ages and humans with colored and natural hair had no statistically significant differences in TCS concentration levels. The obtained results have clearly indicated that people living in northeastern Poland are exposed to TCS to a large degree, and hair analysis, despite some limitations, is a suitable method for TCS biomonitoring in humans.

## 1. Introduction

**Figure 1:**
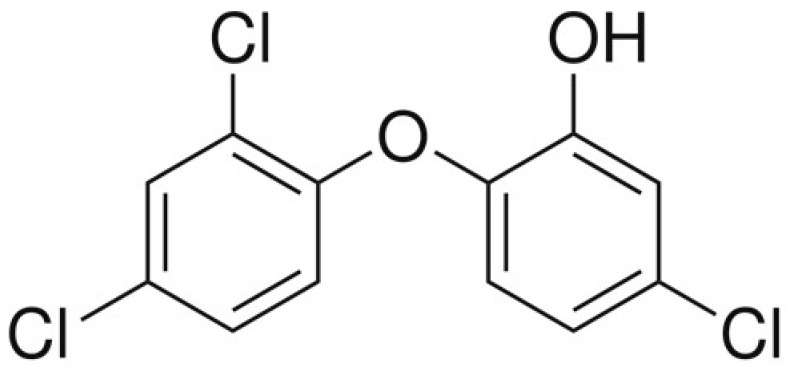
Structure of triclosan (TCS).

Triclosan (2,4,4′-trichloro-2′-hydroxy diphenyl ether—TCS) (Figure 1) is an organic substance belonging to a group of chlorinated phenols [1]. Due to its well-known, strong antibacterial and antifungal properties, TCS has commonly been used since the 1970s in the production of a wide range of consumer products, including detergents, soaps, disinfectants used in medicine, toys, toothpaste, deodorants, clothing, towels and much more [1,2,3]. The widespread use of TCS has resulted in the release of this compound into the environment. Up to now, TCS has been found in surface and tap water, soil, dust and food, and the concentration levels of this substance clearly depend on the part of the world where the studies were carried out [1,4,5,6,7], which may result from the industrialization of the various regions studied and/or differences in the use of triclosan in different regions of the world. It should be pointed out that the presence of TCS has also been described in relatively non-polluted areas away from human habitation, such as the Antarctic coast, which undoubtedly proves the global impact of human activity on the natural environment [8]. 

Previous studies have shown that TCS may penetrate into living organisms. Mainly, it penetrates the digestive tract and skin, but it can also enter the organism through the mucosal membrane of the oral cavity and lungs [1,9,10,11]. The majority of investigations have described the presence of TCS in urine samples [7,12,13,14], but this substance has also been noted in blood serum [15], hair [16,17], nails [18], breast milk [19] and some tissues including the liver, adipose tissue and/or nervous system [20]. The presence of TCS has also been noted in domestic and wild animals [21,22]. In turn, in experimental animals exposed to TCS, high concentration levels of this substance have been described in the liver, gallbladder and epididymis [23,24].

For years, TCS has been regarded as a neutral substance for the living organism. However, the latest observations have described that this substance adversely affects various internal organs and systems. Therefore, TCS is considered to be a potential endocrine disruptor. It is known that TCS affects the immune system, leading to changes in the synthesis of some cytokines [25,26,27]. It also impairs the functioning of the male and female reproductive system [13,28,29], as well as affecting other internal organs, including the heart, liver, digestive tract and others [30,31,32]. Moreover, TCS can cross the blood–brain barrier [33] and shows neurotoxic properties [34]. Some studies have reported correlations between exposure to TCS and the risk of autism, disturbances of social behaviors [35,36] and asthma [37], and the role of this substance in neurodegenerative diseases cannot be excluded [38]. Other investigations have described that TCS exposure may lead to obesity [14], diabetes [39] and cancer development [40].

Until recently, human exposure to organic xenobiotics that pollute the environment has been mainly determined by analyzing urine and/or blood samples, but in recent years, the analysis of hair samples has become more and more important [16,41,42,43]. Hair collection is easy, painless and stress-free. Samples may be stored and shipped easily and cheaply, without the need for freezing. Simultaneously, previous investigations concerning the evaluation of human exposure to xenobiotics have reported that the method is appropriately sensitive, and the obtained results are reproducible and well reflect the long-term human exposure to these substances [16,17,41,44].

Nevertheless, it should be pointed out that the use of hair samples in the biomonitoring of xenobiotics is a relatively new method, and not all aspects connected with this issue are clear. According to the best knowledge of the authors, until now, only three investigations have described the presence of TCS in human hair samples. In one case, samples were collected in Germany [45], and in two studies they were collected in Greece [16,17]. 

The main matrix in studies on human exposure to TCS are the urine samples. However, although urine analysis is invaluable in toxicology and well reflects TCS concentration levels in the organism, it does not explain all aspects connected with the evaluation of exposure to TCS and the distribution of this substance in the organism. This is because TCS levels in the urine change frequently due to daily excretion. It is known that orally administered TCS is rapidly distributed in the organism through the blood, but the major fraction of this substance is extracted within 24 h after administration, and even more than 80% of TCS is extracted during the first 4 days after exposure [11]. Therefore, urine sample analysis has some limitations and does not seem very appropriate to study long-term exposure to TCS. For example, in humans exposed to TCS periodically, TCS concentration levels in urine samples (collected in a period without exposure to TCS) may not reflect the true degree of exposure. In hair, contrary to the urine samples, TCS may accumulate and can remain inside the hair until it falls. Therefore, hair sample analysis is appropriate to study the long-term exposure, even in cases when this exposure is only periodic. Moreover, hair sample analysis monitors both internal (through digestive tract) and external (direct penetration into the hair from the external environment) human exposure to TCS, which is also important in the comprehensive assessment of human exposure to TCS.

Therefore, the aim of this study was to evaluate human exposure to TCS using hair samples collected from adult citizens of Olsztyn, a city in northeastern Poland, as well as correlating those samples with gender, age, hair coloring and TCS concentration levels. The study supplements knowledge both about human exposure to TCS in the world and hair analysis as a method used in xenobiotic toxicology.

## 2. Materials and Methods

### 2.1. Materials

TCS and ammonium acetate (≥98%) were purchased from Sigma-Aldrich, methanol (LC-MS grade) from Honeywell Riedel de Haën (Seelze, Germany), acetonitrile (LC-MS grade) from Fisher Chemical and phenobarbital-d5 from Isotec Inc. (Miamisburg, OH, USA). Ultrapure water was produced by Merck’s Direct-Q 3UVwater purification system (Darmstadt, Germany). 

### 2.2. Sampling

Head hair samples (about 2 g) were collected in 2021 from 30 volunteers (15 males and 15 females) who were residents of Olsztyn, a city with about 170,000 residents in northeast Poland. Volunteers were aged 22–67 (mean 39.2 ± 13.85) and did not report any chronic medical conditions and/or continued medication. Moreover, basic information, such as gender, age and if the hair was dyed or not, was collected from volunteers. Information about volunteers included in the study is shown in Table 1. All volunteers were informed about the planned study and gave their consent to the sampling procedure. All activities in this study were made in accordance with the agreement of the Bioethical Committee at the School of Medicine of the University of Warmia and Mazury in Olsztyn (Poland) (No. agreements: 27/2017 and 5/2021). Hair samples were collected from a place above the nape of the neck, closest to the scalp. Immediately after collection, they were wrapped in aluminum foil and stored in the dark at room temperature until analysis.

### 2.3. TCS Extraction

Hair samples were washed in ultrapure water (two times) and in methanol (two times) to remove external contamination. After washing, samples were dried and cut into small pieces. The extraction process was made in accordance with the method previously described [17,46]. In brief, 100 mg of hair samples were placed into glass screw tubes and treated with 2 mL of methanol. Samples were subjected to extraction in an ultrasonic water bath for 4 h with periodic mixing with a vortex system. After centrifugation, the extract was put in glass tubes and evaporated to dryness under nitrogen steam at 35 °C. Then, 100 μL of methanol was added to residues and the solution was transferred into 2 mL vials for liquid chromatography–mass spectrometry (LC–MS) analysis. The selected *m*/*z* ions for the monitoring of triclosan were 286.95, 289.00 and 291.00. The elution gradient program started at 15% solvent B (1 min), 95% solvent B (20 min) and finally at 15% solvent B for 4 min. Under the above chromatographic conditions, the retention time of TCS was 22.03 min.

### 2.4. Analysis

During the present study, one measurement was made per hair sample. The analysis was performed with a liquid chromatography–mass spectrometry system (LC–MS 2010 EV, Shimadzu, Kioto, Japan), and the separation of TCS was made on a Discovery C18 column (250 mm, 4.6 mm, 5 μm) (Supelco, Bellefonte, PA USA) at 30 °C. A gradient of 5 mM ammonium acetate (solvent A) and acetonitrile (solvent B) were chosen for the analysis at 0.6 mL/min. Atmospheric pressure chemical ionization (APCI) combined with a quadrapole mass filter in a selected ion monitoring (SIM) negative mode were used for monitoring the aforementioned substance. 

### 2.5. Method Validation

To ensure the efficacy of the method used in the present study, analytical parameters were checked. Standard solutions of TCS were performed in concentrations of 0, 50, 100, 250 and 500 ng/mL. Linearity of the standard solution responses was found to be 0.9998. Moreover, spiked samples were performed in concentrations of 0, 50, 100, 250 and 500 pg/mg, and linearity was found at 0.9937.

Limit of detection (LOD) and limit of quantification (LOQ) were calculated using the signal to noise ratio. Recovery, accuracy and inter-day precision (%RSD) of the method were examined using 3 repeats of spiked samples (n = 3) for each of the above-mentioned tested concentrations (50, 100, 250 and 500 pg/mg) (Table 2).

### 2.6. Statistical Analysis

A non parametric Mann–Whitney test—GraphPad Prism version 9.2.0 (GraphPad Software, San Diego, CA, USA)—was used in the statistical analysis during the comparison of two groups of volunteers (males versus females, younger volunteers versus older ones, persons with colored hair versus persons with non-colored hair). The differences were considered statistically significant at *p* < 0.05.

## 3. Results

**Figure 2:**
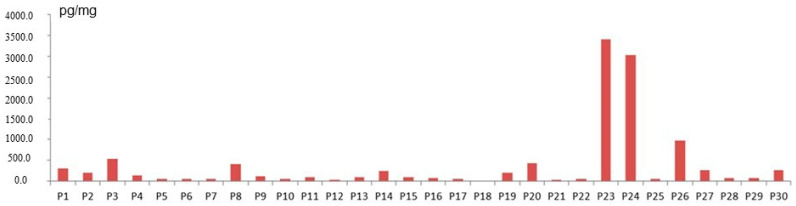
Concentration levels of TCS in particular subjects included in the study.

TCS was found in 96.7% of samples tested. The concentration levels of this substance in samples from particular volunteers were very different and fluctuated from 37.9 pg/mg to 3386.5 pg/mg. The mean concentration level of TCS in the present study was 402.6 (± 803.6) pg/mg and the median was 103.3 pg/mg. Concentration levels of TCS noted in the present study are presented in Figure 2.

**Figure 3:**
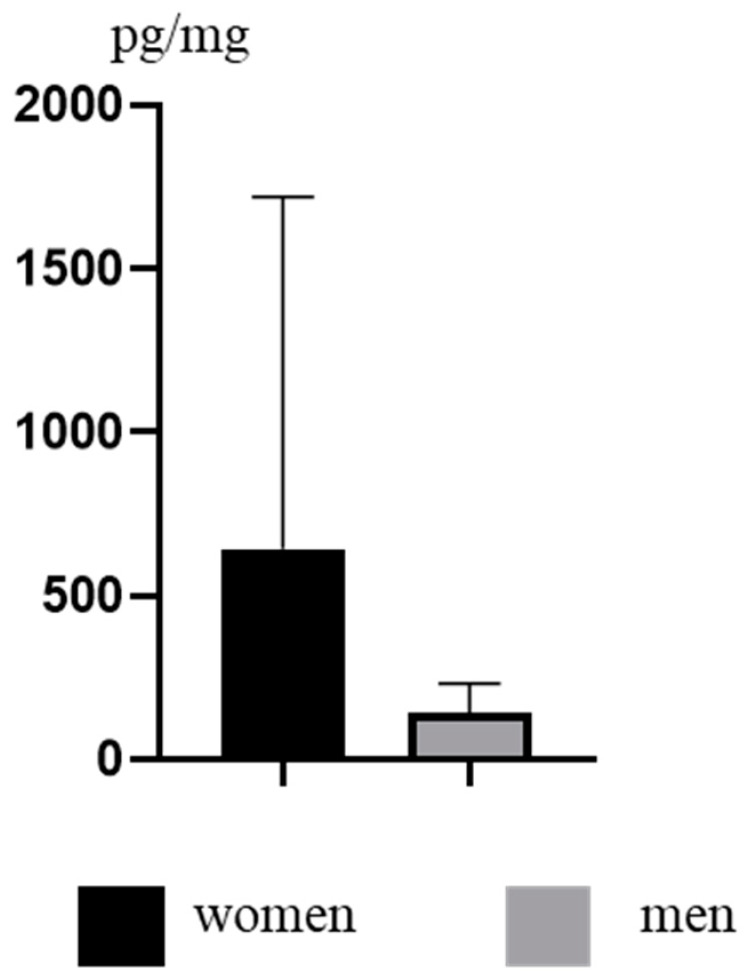
Mean concentration levels (±SD) of TCS in men and women.

During the present study, concentration levels of TCS in women and men were different. In women, the concentration levels of this substance fluctuated from 37.90 pg/mg to 3387 pg/mg (mean 643.6 ± 1075 pg/mg). In men, the values were lower and fluctuated from 51.80 pg/mg to 318.6 pg/mg (mean 144.4 ± 88.40 pg/mg) (Figure 3). However, differences between genders were not statistically significant.

**Figure 4:**
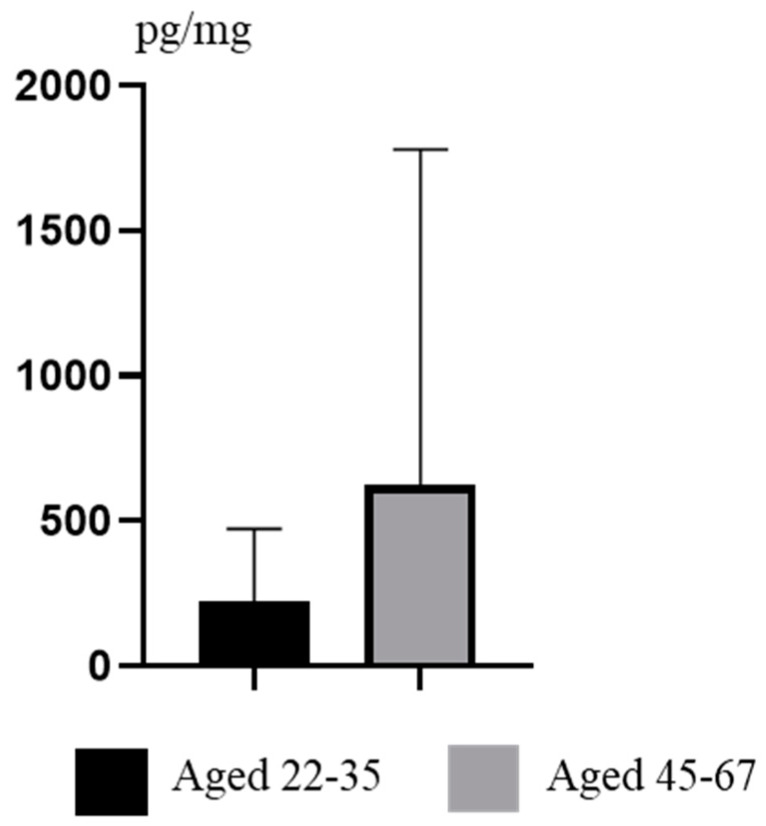
Mean concentration levels (±SD) of TCS in persons at the age of 22–35 and 45–67.

Some differences in concentration levels of TCS were also visible between volunteers at the age of 22–35 and persons at the age of 45–67 (Figure 4). In the first group, values fluctuated from 37.90 pg/mg to 984.6 pg/mg (mean 222.7 ± 249.4 pg/mg). In older people, values were higher and fluctuated from 49.30 pg/mg to 3337 pg/mg (mean 624.1 ± 1154 pg/mg). Similar to intergender differences, differences between groups of persons of various ages were also not statistically significant.

**Figure 5:**
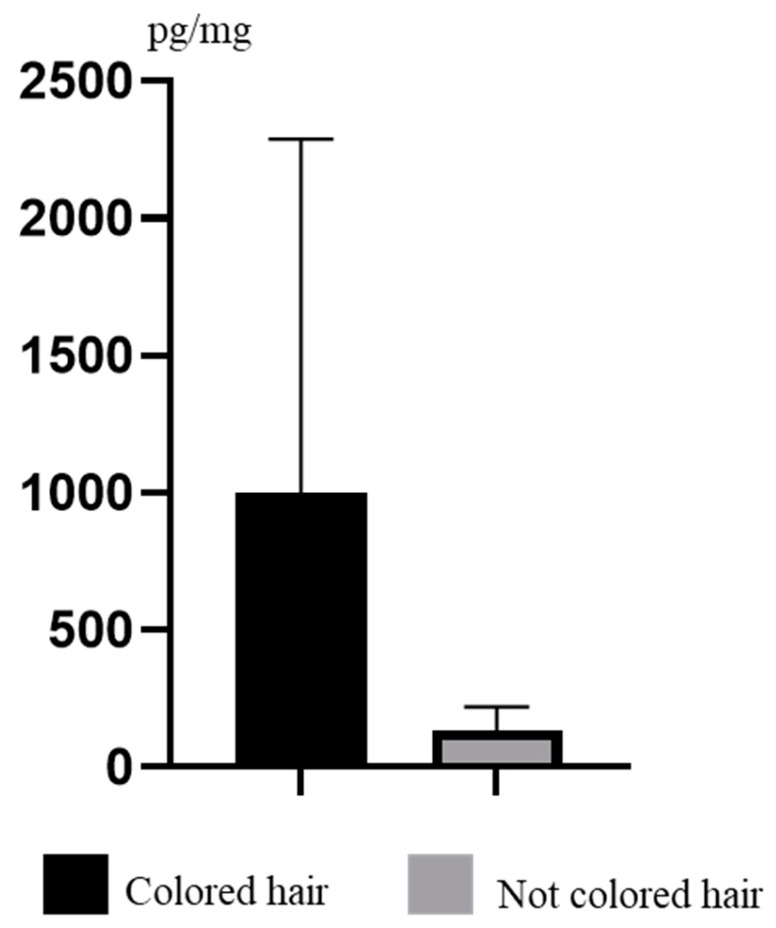
Mean concentration levels (±SD) of TCS in persons with colored and non-colored hair.

During the present study, the comparison of TCS concentration levels in volunteers with colored and non-colored (natural) hair was also done. In persons with colored hair (only women, all men included in the study had natural hair), the concentration levels of TCS fluctuated from 49.30 pg/mg to 3387 pg/mg, with a mean value of 997.9 ± 1290 pg/mg. In persons with natural hair, the values were smaller and ranged from 37.90 to 318.6 pg/mg, with a mean value of 134.7 ± 85.33 pg/mg. However, there were no statistically significant differences between these groups (Figure 5).

## 4. Discussion

During the present study, TCS was found in a high percentage (96.7%) of samples analyzed, which proves a high degree of human exposure to this substance. On one side, such exposure is connected with the common use of TCS in the production of personal care products, cosmetics, medicaments and numerous other everyday objects [1,2]. On the other side, it may also indicate a relatively high level of environmental pollution with triclosan, which has been confirmed by previous studies, which described the presence of TCS in water, indoor dust, soil and food [2,3,7]. Moreover, during the present investigation, highly visible differences were noted between particular volunteers included in the study; this observation is in agreement with previous studies concerning human exposure to TCS using various matrices (Table 3). 

Such visible differences between minimal and maximal TCS concentration levels in volunteers living in the same neighborhood strongly suggest that human exposure depends on various factors. In light of previous studies, the degree of human exposure to TCS depends not only on the scale of the use of cosmetics and personal care products and the industrialization of the region where the samples were collected, but also may be connected with many other factors, including diet, profession, type of clothing, medication use, lifestyle and even dental fillings and socioeconomic status [1,2,56]. In spite of numerous factors affecting TCS concentration levels in the human organism and large differences in these levels between particular regions of the world, clear differences in TCS content depending on the matrix studied have also been visible (Table 3). The lowest TCS concentrations have been noted in breast milk, which may suggest a relatively low TCS penetration capacity from the blood to the mammary gland. The higher, but very varied levels of TCS have been observed in the blood and urine. On the one side, this fact confirms that TCS is distributed throughout the body through the blood and urine is a main pathway for TCS excretion, and on the other side, that TCS concentration levels in the blood and urine undergo short-term changes. Interestingly, maximum concentration levels of TCS in the hair are clearly higher than maximum values in the urine or blood (Table 3). The reason for such a situation is unknown so far, but it is probably connected with the fact that hair analysis includes not only “internal exposure”, where TCS reaches the hair root through the blood, but also “external exposure”, where there is direct penetration of TCS from the environment into the hair. However, this hypothesis still needs to be confirmed by comprehensive studies of TCS concentration levels in different matrices taken from the same people. 

Such comprehensive studies are also indispensable to clarify one more very important issue related to the presence of TCS in the human hair. Namely, it is about the possibility of bioavailability of TCS from the hair (through hair follicles) into the systemic blood circulation (and then into urine samples) and the associated risk of higher human exposure to this substance. If the degree of this bioavailability was established, then it would allow the updating of human biomonitoring assessment values (HBM values) for TCS. There are two kinds of HBM values: the HBM I value—at which and below which there is no risk of adverse health effects—and the HBM II value—at which and above which adverse health effects are possible [57]. Currently, only an HBM I value has been assigned for TCS in the urine: 2 mg/L in children and 3 mg/L in adults [57]. Unfortunately, there is no information about the possibility of TCS penetration from the hair into the blood as of yet, although it cannot be ruled out since some other substances have such an ability [58,59].

The exact determination of the source of human exposure to TCS in Poland poses considerable difficulties due to the fact that research on the presence of this compound in everyday products, as well as in food or the Polish natural environment, is very limited. All previous studies concerning this issue in Poland are presented in Table 4.

Due to the fact that previous studies on human exposure to TCS in Poland have been performed on urine samples, it is difficult to compare the present results with previous observations. Moreover, between particular studies conducted in Poland on urine samples, clear differences have been visible (Table 4). This is further evidence that the concentration of TCS in the urine depends on many factors and is subject to short-term changes. The comparison of TCS concentration levels observed in previous studies with the present results is even more difficult, as previous studies were not carried out near Olsztyn but in other regions of Poland, and, as it is known, exposure to TCS depends on the region in which the observations are carried out (Table 3). This is most likely related to environmental pollution and differing levels of personal care product usage containing TCS. However, in spite of the fact that results have been obtained in various matrices, the present results are clearly higher than results obtained in previous studies on human exposure to TCS in Poland, which is in line with the trend also observed in other regions of the world (Table 3) and may be related to the above-mentioned fact that hair analysis includes both internal and external exposure to TCS.

It should be pointed out that the previous studies described TCS concentration levels mainly in the urine, and the hair sample analysis for the presence of TCS is a relatively new method. Until recently, only three previous publications have shown TCS in human hair samples (Table 3). Comparing the results obtained in the present investigation with previous studies on human hair (Table 5), it should be concluded that the median value (103 pg/mg) obtained in the present study is significantly higher than the median observed in adult women (61.6 pg/mg), noted in Greece by Karzi et al. [17]. In turn, compared to the median value obtained in the present study with another study performed in Greece [16], it can be concluded that it is higher than the median in children (48.8 pg/mg) but lower than the median in adults of both genders (181.6 pg/mg). 

The third previous publication described TCS in human hair collected in Germany (Table 3) and was performed on only four volunteers and did not present median or mean values [45]. These differences confirm previous observations performed on other matrices, which have clearly shown correlations between TCS concentration levels in the human organism and the part of the world where the studies were made (Table 3). These correlations have arisen as a consequence of various environmental factors mentioned above.

During the present study, statistically significant differences in TCS concentration levels were not found between males and females, although the values of concentration levels in women were higher than those noted in men. Although it is generally believed that a higher concentration of TCS in women may be connected with the fact that they use different types of cosmetics and personal care products containing this substance more often and to a greater extent than men, the previous studies have often reported contradicting results. For example, Allmyr et al. [15] conducted studies in Australia showing that the blood serum of males had higher concentration levels of TCS than females. In turn, studies performed in China showed higher concentration levels of TCS in the urine found in females [63], and other investigations performed on the urine samples (conducted in Belgium and Denmark) did not show statistically significant differences in TCS concentration between genders [64,65]. In previous studies performed on human hair samples, the results were very similar to those noted in the present study because higher levels of TCS have been noted in females than males but without statistical differences [16].

Intragender differences between TCS concentration in humans are difficult to establish due to a wide range of factors having an impact on exposure to TCS. However, experiments on animals strongly suggest that intragender differences may be connected not only with more frequent use of cosmetics by women, but also with various metabolic processes in males and females. Namely, it has been shown that the absorption of TCS is greater in female than in male mice [22], and so females are more exposed to TCS. 

A comparison between groups of younger (aged 22–35) and older (45–67) volunteers performed in the present study have shown that TCS concentration levels were higher in the second group, but the differences were not statistically significant. In light of previous studies, it is known that samples collected from adult humans have shown higher TCS concentration levels than samples from children in the urine [56,64] and hair samples [16]. Moreover, it has been found that TCS concentration levels in blood serum [15] and urine [56] are the highest in people aged 30–45 years, and in older humans they fall again. The mechanisms of such a situation are not clear, but it can be assumed that it is connected with lifestyle and a high degree of cosmetics usage and personal care products containing TCS by people aged 30–45 years in comparison to both younger and older persons.

The analysis of human hair samples to evaluate endocrine disruptor concentration levels, despite its many advantages, has some limitations. One of them is the possible influence of hair coloring on the results. During the preparation of samples for analysis, a special emphasis is placed on removing any contamination from the hair surface. However, it is commonly known that hair dyes, which often contain oxidative substances, impact the hair structure, which may lead to a change in structural integrity and therefore easier penetration of substances from the environment into the hair [66,67,68]. Previous studies are not unequivocal. For example, some authors have described the influence of hair coloring on the concentration of parabens in the hair samples [41], whilst other researchers have not found differences in the concentration of these substances between humans with colored and natural hair [69,70]. Studies on the influence of hair coloring on TCS concentration levels have not been performed until now. Although this study has not shown statistically significant differences in TCS concentration levels between volunteers with colored and non-colored hair, relatively clear higher concentrations in people with colored hair do not allow us to exclude the influence of hair coloring on TCS levels in hair samples. Therefore, further studies are needed to clarify this issue. Moreover, the present investigation was performed on a relatively small number of samples, which is undoubtedly a limitation of this research.

Another limitation connected with hair sample analysis is that the chemicals penetrate the hair in two ways: internally from blood to the hair bulb and externally directly from the environment to the inside of the hair. During analysis, the total concentration level in the hair is determined and it is impossible to separate the internal and external penetration. Moreover, it should be remembered that substances accumulate in the hair and are in the hair until the hair falls out, contrary to blood serum and urine in which the levels of substances may change in quick succession. Therefore, hair analysis is a method suitable for studies on long-term exposure to environmental pollutants, whilst investigations on urine and blood samples are more appropriate for the evaluation of short-term changes in exposure.

Of course, this study also has some other limitations and did not answer many questions related to the investigation of human exposure in Poland to TCS by hair sample analysis. For example, due to the relatively small number of volunteers and the fact that all volunteers included in the study did not declare chronic diseases, it is difficult to determine if TCS exposure levels found in this study constitutes a risk factor for human health. On the other hand, in light of numerous previous studies [27,28,29,30,31,37], which have reported multidirectional influences of this substance on living organisms, it seems highly probable that a higher degree of exposure to TCS may be associated with a greater risk of disturbances in the functioning of the human organism. Moreover, during the present study, the questions asked of the volunteers concerned only basic issues such as age, gender and hair dye, while large differences between particular volunteers strongly suggests that other factors may also influence the degree of exposure to TCS. It should be underlined that to define those factors, which may include, among others, occupation, frequency of use and type of cosmetics, diet and lifestyle, a comprehensive study on a larger group of volunteers and a more detailed questionnaire are necessary. Nevertheless, the present study may be treated as a pilot study, which clearly showed that, on the one hand, in northeastern Poland, there is a risk of exposure to TCS, and on the other hand that hair analysis may be used to evaluate this exposure. This study may also be the starting point for further studies on this issue.

## 5. Conclusions

To sum up, the present study has shown that humans in Poland are exposed to TCS to a significant degree and that hair analysis may be used to bio-monitor this substance in humans. Large differences in TCS concentration levels between particular volunteers may suggest that exposure to this substance depends on various factors. Statistically significant correlations between TCS concentration levels and gender, age and/or hair coloring have not been observed. However, it cannot be clearly ruled out that hair coloring does not affect the concentration levels of TCS in the hair, which may limit the use of this method to some extent. To explain all aspects connected with the use of hair sample analysis to bio-monitor TCS in humans, further comprehensive studies on a larger population are necessary.

## Figures and Tables

**Table 1:** Data of volunteers included in the study.

No.	Age	Gender	Hair Color	Hair Coloring
1	46	Male	Black	No
2	45	Male	Black	No
3	28	Female	Brown	Yes
4	23	Male	Black	No
5	32	Male	Black	No
6	34	Male	Black	No
7	50	Female	Blond	Yes
8	53	Female	Black	Yes
9	28	Male	Black	No
10	22	Female	Blond	No
11	27	Female	Black	No
12	24	Female	Brown	No
13	23	Female	Brown	No
14	23	Male	Brown	No
15	61	Male	Black-Gray	No
16	35	Male	Black	No
17	55	Male	Black-Gray	No
18	49	Male	Black	No
19	23	Male	Brown	No
20	22	Female	Black-Brown	Yes
21	56	Female	Brown	Yes
22	55	Female	Brown-Red	Yes
23	48	Female	Brown	Yes
24	46	Female	Black	Yes
25	50	Male	Grey	No
26	31	Female	Brown	Yes
27	27	Female	Brown	No
28	67	Female	White	No
29	34	Male	Black	No
30	59	Male	Black	No

**Table 2:** Validation parameters of the applied methodology.

n = 3	
Mean % recovery	117.0
±SD	18.6
Mean % accuracy	115.6
±SD	24.8
Precision (% RSD)	15.6
±SD	7.3
LOD (pg/mg)	0.7
LOQ (pg/mg)	2.4
r^2^ (spiked curves)	0.9937
r^2^ (standard curves)	0.9998

**Table 3:** TCS concentration levels in humans in light of selected previous studies. Concentrations in ng/mL for liquid samples and in ng/g for solid samples.

Country	Matrix	Method	Range of TCS Concentration	References
Belgium	urine	GC-MS/MS	ND–598.95	[47]
Brazil	urine	LC-MS/MS	<0.5–294	[48]
Canada	urine	LC-MS/MS	<0.008–19.8	[49]
China	urine	LC-MS/MS	<0.003–178	[50]
China	urine	LC-MS/MS	0.08–1600	[12]
Greece	urine	LC-MS/MS	0.08–386	[12]
India	urine	LC-MS/MS	0.08–898	[12]
Japan	urine	LC-MS/MS	0.08–287	[12]
Kuwait	urine	LC-MS/MS	0.26–288	[12]
Saudi Arabia	urine	LC-MS/MS	0.08–34.4	[12]
Slovenia	urine	GC-MS/MS	<0.25–25	[51]
South Korea	urine	LC-MS/MS	0.08–558	[12]
USA	urine	LC-MS/MS	0.21–819	[12]
Vietnam	urine	LC-MS/MS	0.08–27	[12]
Great Britain	blood serum	LC-MS/MS	<0.22–178.75	[52]
China	blood serum	UHPLC-Q-TOF/MS	0.15–217	[53]
Spain	blood serum	GC-MS	<1.4–12 ± 1	[54]
Spain	breast milk	GC-MS	<1.3–0.70 ± 0.05	[54]
Sweden	breast milk	GC/ECNI/MS	<0.018–0.32	[55]
China	nails	UHPLC-MS/MS	ND–5049.19	[18]
Germany	hair	GC-MS	276–1870	[45]
Greece	hair	LC-MS	3.6–8564.9	[16]
Greece	hair	LC-MS	8.8–8070.2	[17]
Poland	hair	LC-MS	37.9–3386.5	This study

GC/ECNI/MS—Gas chromatography in combination with electron capture negative ion mass spectrometry; GC–MS/MS—gas chromatography/tandem mass spectrometry; GC-MS—Gas chromatography/mass spectrometry; LC-MS—Liquid chromatography/mass spectrometry LC-MS/MS—Liquid chromatography/tandem mass spectrometry; ND—not detected; UHPLC-MS/MS—Ultra-high performance liquid chromatography/tandem mass spectrometry; UHPLC-Q-TOF/MS—Ultra-high-performance liquid chromatography/quadropole time-of-flight/mass spectrometry.

**Table 4:** TCS in Poland in light of previous studies. Concentrations in ng/mL for liquid samples and in ng/g for solid samples.

Matrix	Method	TCS Concentration Levels	References
Human urine: women	GC–MS/MS	0.3–1677.68	[13]
Human urine: women	GC–MS/MS	0.3–265.17	[60]
Human urine: men	GC–MS/MS	<LOD–789.20	[28]
Surface water	ELISA	0.021–2.457	[61]
Surface water	LC-MS/MS	0.0446–0.2715	[52]
Wastewater	LC-MS/MS	0.0498 ± 0.0007–6.7217 ± 0.0068	[62]
Human hair	LC-MS	37.9–3386.5	This study

GC–MS/MS—gas chromatography/tandem mass spectrometry, ELISA—enzyme-linked immunosorbent assay, LC-MS/MS—liquid chromatography/tandem mass spectrometry, LC-MS—liquid chromatography/mass spectrometry, LOD—limit of detection.

**Table 5:** Median values of TCS concentration levels in human hair studied with the liquid chromatography/mass spectrometry method (pg/mg).

Country	Groups of Volunteers	Median Value of TCS Concentration Levels	References
Greece			
	Children of both gender	48.8	[16]
	Adults of both gender	181.6	[16]
Greece			
	Adult pregnant women	61.6	[17]
Poland			
	Adults of both gender	103	This study

## Data Availability

Not applicable.
